# Removal of amoxicillin from wastewater by self-made Polyethersulfone membrane using nanofiltration

**DOI:** 10.1186/s40201-014-0127-1

**Published:** 2014-10-22

**Authors:** Ahmad Moarefian, Hossein Alizadeh Golestani, Hooman Bahmanpour

**Affiliations:** Department of Chemical Engineering, College of Science, Shahrood Branch, Islamic Azad University, Shahrood, Iran; Department of Chemical Engineering, Quchan Branch, Islamic Azad University, Quchan, Iran; Department of Environment, College of Science, Shahrood Branch, Islamic Azad University, Shahrood, Iran

**Keywords:** Nanofiltration, Polyethersulfone membrane, Amoxicillin removal, Wastewater treatment

## Abstract

The present study investigated the performance of a self-made nanofiltration (NF) membrane for the removal of antibiotics from wastewater under changing operating conditions such as pH, initial feed concentration, operating pressure, and temperature. Amoxicillin (AMX) was used as one of the commonly prescribed antibiotics. A self-made NF membrane containing Polyethersulfone (PES), and Polyvinylpyrrolidone (PVP) was modified with Polyethylene glycol hexadecyl ether (Brij®58) surfactant. The self-made membrane was characterized by water contact angle, zeta potential, ATR-FTIR spectroscopy, and scanning electronic microscope (SEM). The obtained results showed that the AMX rejection and permeation flux by the self-made membrane varied from 56.49% to 99.09% and from 15.14 L/m^2^h to 110.29 L/m^2^h, respectively. The AMX rejection decreased at a higher level of initial feed concentration while other operating parameters such as pH, operating pressure, and temperature had a negligible effect on the removal of AMX from wastewater by the self-made NF membrane. The highest removal rate was achieved under conditions of pH 9.0, a temperature of 298 K, an operating pressure of 2 MPa, and an initial feed concentration of 20 ppm. According to the research findings, the self-made NF membrane is recommended for the removal of AMX to a considerable extent at low initial feed concentrations.

## Introduction

The occurrence of antibiotics as emerging containment substances in aquatic environments has always been a cause of concern due to the destructive potential on ecosystems. Prolonged exposure to trace levels of antibiotics leads to the selective proliferation of antibiotic-resistant bacteria and long-term adverse effects on ecosystems and human health [1-7]. However, antibiotic residues have been detected in surface water, ground water, and the final effluent of wastewater treatment plants (WWTPs), even at low concentrations in the range of nanograms/micrograms per liter [8-10].

Although Amoxicillin (AMX) is used to treat a number of infections, however, it is suspected of direct toxic effects on certain aquatic organisms [11-15]. Moreover, amoxicillin-resistant pathogens, such as *Klebsiella pneumoniae*, and *Bacteroides spp* [16] in aquatic environments are a potential health threat and make water aesthetically pleasing. According to above-mentioned details, antibiotic residues such as AMX should be removed from aqueous matrices. Common physical and chemical treatment methods are not suitable for the removal of large quantities (mg/L) of pharmaceutically active compounds (PhACs) from wastewater [17,18]. Accordingly, these methods are not efficient for treatment of PhACs [19].

Efficient removal of the polar PhACs can only be ensured using more advanced methods such as ozonation, advanced oxidation processes (AOP), activated carbon, or membrane filtration [19]. However, high cost of equipment and maintenance, as well as energy supply are of disadvantages of the ozonation technique [6]. Capital intensive required to quenching of excess peroxide for some applications [20] rejects the use of AOP. Although granular activated carbons (GACs) are applicable adsorbents, however, they are costly and their regeneration is difficult [6]. The removal efficiency by activated carbon is low for PhACs having low log K_ow_ values (e.g. AMX by log K_ow_ = 0.87 [21]) and low electrical charges [19].

Membrane technologies such as nanofiltration (NF) and reverse osmosis (RO) have extensively been used for municipal wastewater treatment purposes to ensure the quality of municipal effluents and wastewater reuse [18]. Pressure-driven membrane processes, particularly NF and RO have been the centre of attention in the past few years especially for treatment of drinking water [22,23].

NF is a relatively recent membrane filtration process being used up rapidly [24]. NF membranes are mainly used for the removal of dissolved PhACs from water matrices [22,25]. Compliance of molecular mass (MW) of PhACs ranges between 200 and 1200 Da, with the molecular mass cutoff (MWCO) of NF membranes [24], NF seems to be an efficient technique for the removal of antibiotics from contaminated water [26].

Most NF membranes are charged by the dissociation of surface functional groups such as carboxylic or sulfonic [6,25]. Negatively charged NF membranes are widely used because they can selectively pass or reject the ions from feed solution through the electrostatic interaction between ions and membrane surface [6,25].

Moreover, different rejection mechanisms have been proposed to NF process, which include, molecular sieving (steric hindrance), Donnan exclusion (electrostatic interaction between charged solutes and membrane-attached charges), and dielectric exclusion (interaction between ions and the polarized charge) [25]. In spite of many methods on characterization of NF membranes, the transport mechanisms of solutes through membranes are not completely understood [25].

This research was conducted to investigate the performance of a self-made membrane for removal of AMX from synthetic wastewater under varying operating conditions.

## Materials and methods

### Materials

**Figure 1:**
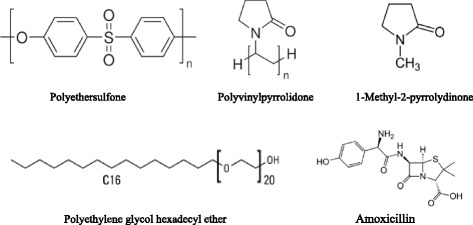
**Chemical structure of PES, PVP 40, NMP, Brij®58 and AMX.**

Polyethersulfone (Mw = 58000 g/mol) provided by BASF Co. was used to prepare membrane casting solution. Pure AMX (Mw = 365.40 g/mol) was prepared from Dana Pharmacy Co. (Tabriz, Iran). Polyvinylpyrrolidone (Mw = 40000 g/mol), 1-Methyl-2-pyrrolidinone (NMP) as a polymer solvent, and Polyethylene glycol hexadecyl ether (Brij®58) as a non-ionic surfactant (HLB = 15.7) were purchased from Sigma Aldrich Co. (USA). N,N-dimethyl-p-phenylenediamine, potassium hexacyanoferrate (iii), NH_3,_ and NaOH were bought from Merck Co. (Germany) to determine AMX content in feed and permeate flow. Molecular structure of PES, Brij®58, PVP, NMP, and AMX is shown in Figure 1.

### Membrane preparation

The self-made membrane was prepared by phase inversion method using optimal amounts of PES, PVP, and Brij®58 surfactant. The Brij®58 was used as a non-ionic surfactant to modify the PES membrane. The casting solution was prepared by dissolving 1.143 g of Brij®58 in 13.155 mL of NMP, then 4.000 g of PES and 0.381 g of PVP was gently added to residue materials as a pore former and stirred overnight at 300 rpm. After preparing a homogeneous solution, the dope was kept at room temperature for about 24 h until the air bubbles were removed. The homogenous solution was casted onto a glass substrate hovering at a height of 200 μm using a film applicator at room temperature without evaporation. Then, the solution was transferred to a deionized water bath for immersion precipitation at 273 K and allowed it to stand for 5 minutes.

The prepared membrane was stored in distilled water for 24 h to allow the water soluble components to be leached out. Finally, the membrane was dried by two filter papers under very low uniform pressure.

### Synthetic wastewater

The synthetic wastewater with initial feed concentrations of 20 and 400 ppm was prepared by dissolving 0.2 and 4 g of AMX in 10 L distilled water. In order to investigate the effect of pH on the performance of the self-made membrane for the removal of AMX, all the experiments were conducted at pH values of 5, 7.0, and 9.0. The pH of feed solution was about 5. 0.1 M (0.1 N) sodium hydroxide (NaOH) was added to the synthetic wastewater in order to adjust the pH at 7.0 and 9.0.

### Membrane characterization

#### Contact angle

In order to evaluate membrane hydrophilicity, static contact angle between water and the membrane was measured directly using an OCA 15 Plus (Data Physics Instruments, Germany). Deionized water was used as a probe liquid in all measurements. All contact angle measurements were made using deionized water drops of 4 μl. To minimize experimental errors, for each sample, the contact angle was measured at 4 random locations and then, the average value was considered.

#### Zeta potential

To determine electrical charge over the membrane surface, zeta potential was determined by streaming potential measurements using Electro Kinetic Analyzer (EKA 1.00, Anton-Paar, Swiss) equipped with a plated sample cell. Zeta potential was measured in a 0.001 M KCl solution. The measurements were carried out at 298 K in KCl solution (0.001 M) with polymethyl methacrylate (PMMA) plate. Zeta potential was measured at pH values of 5.0, 7.0, and 9.0.

#### Morphologic study

The morphology of the self-made membrane was studied by an electronic microscope (EM 3200, KYKY, China). For this, a sample of the membrane was frozen in liquid nitrogen and then fractured. After gold sputtering, it was examined by an electron microscopy at 20 kV.

#### ATR-FTIR spectroscopy

In order to ensure the presence of Brij®58 surfactant in the structure of synthetic membrane, the ATR-FTIR spectroscopy was used. The Fourier transform infrared (FTIR) spectrum of the membrane was recorded in the range between 400 and 4000 cm^−1^ using attenuated total reflection (ATR) technique by Nicolet IR 100 FTIR spectrometer (Thermo, USA).

### Experimental set-up

**Figure 2:**
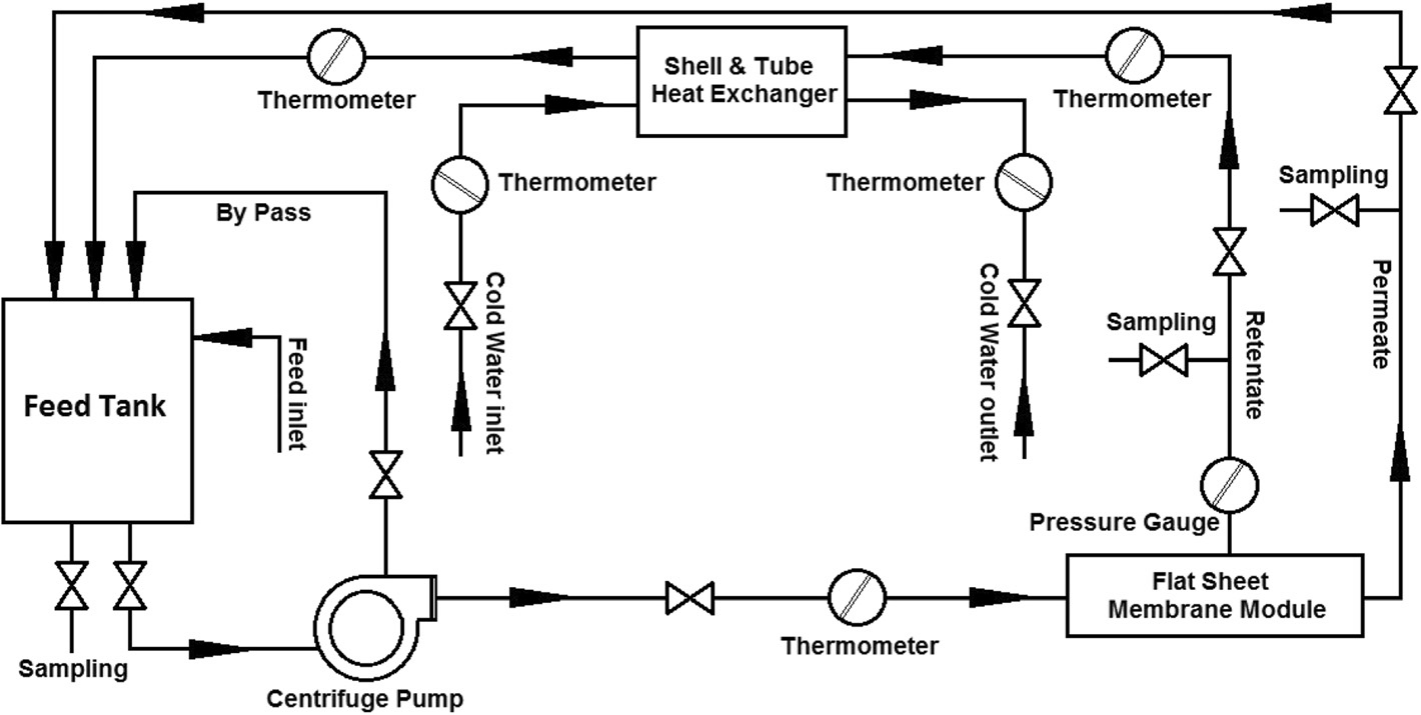
**Schematic diagram of the NF set-up.**

NF experiments were conducted in a consecutive lab-scale filtration equipped with a cross-flow permeation cell with an effective filtration area of 6.936 × 10^−3^ m^2^ supported by a porous stainless steel disc. Details of the permeation cell and NF set-up are depicted in Figure 2. The temperature of feed solution was maintained at 298, 308, and 318 K by a shell and tube heat exchanger. The operating pressure can be varied from 0.5 to 2 MPa. Pressure was adjusted by backpressure and bypass valves. Retentate and permeate streams were directed back into the feed tank in a closed cycle, which makes the feed concentration approximately constant.

### Membrane performance

The membrane was cut into appropriate size required to fix up the NF membrane cell and then pressurized with distilled water at 2 MPa for 1 h. After compression, the pressure was dropped to the operating pressure level of 0.5, 1, and 1.5 MPa. Subsequently, the pure water flux (PWF) was collected for 1 h and calculated using the Eq. (1).

1 $$ \mathrm{P}\mathrm{W}\mathrm{F} = \mathrm{Q}/\mathrm{A}\ \Delta \mathrm{t} $$

Where; PWF = pure water flux (L/m^2^h), Q = quantity of permeate (L), A = active membrane area (m^2^), and ∆t = sampling time (h).

After filtration of the pure water, the feed tank was emptied and refilled with the synthetic wastewater. The membrane performance was studied in terms of either permeation flux or AMX rejection. The solute rejection was calculated using Eq. (2).

2 $$ \mathrm{R}\ \left(\%\right) = \left[\left(1 - {\mathrm{C}}_{\mathrm{P}}/{\mathrm{C}}_{\mathrm{F}}\right)\right]\times 100 $$

Where; C_P_ and C_F_ are concentrations of the solute in permeate and initial feed solutions, respectively. Concentration of AMX in the permeate stream was determined using N,N-dimethyl-p-phenylenediamine, potassium hexacyanoferrate (iii), and NH_4_OH. The absorbance of samples was measured by T60 UV–Vis spectrophotometer (PG Instruments, England) at the maximum wavelength. The measurement technique relies on the Beer-Lambert Law [27].

## Results and discussion

### Membrane morphology

**Figure 3:**
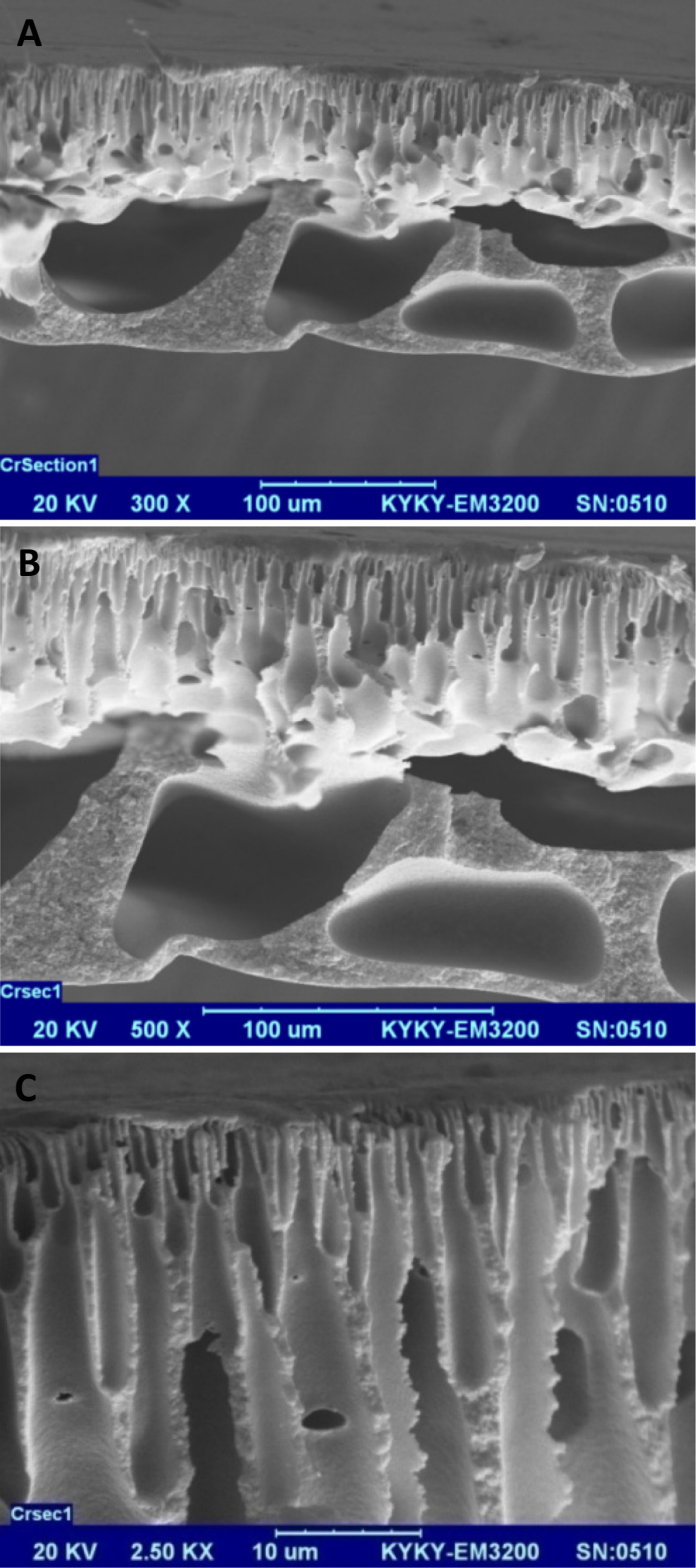
**SEM cross-section of the self-made membrane at magnifications of A) 300×, B) 500×, and C) 2500×.**

Separation of neutral solutes by porous membranes is mainly a function of molecular and pore size distribution [28]. Accordingly, membranes with a higher pore density, surface porosity, and porous sub-layer have a higher permeation flux [29]. In other word, higher porosity provides more pore channels for diffusion that leads to a higher flux. However, the morphologic study can help to predict rejection rate of neutral solutes as well as permeation rate of flux. The SEM images of the self-made membrane demonstrate an asymmetric structure consisting of a dense top-layer and a porous sub-layer (Figure 3). The morphology of the asymmetric synthetic membrane demonstrated finger-like macrovoids developed underneath the dense layer. Higher rate of permeation flux and AMX rejection is expected due to the high-porous sub-layer of the self-made membrane (each pore size ranges between 0.5 and 5 μm) and a dense selective top-layer consisting of nanopores.

### Pure water flux

**Figure 4:**
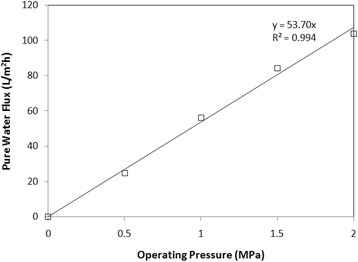
**PWF vs. operating pressure at T = 298 K for PES/PVP/Brij®58 Membrane.**

Figure 4 shows that the PWF tends to increase with increasing TMP. According to which, increased pressure is an effective way to increase permeation flux. Furthermore, there is a linear relation (R^2^ = 0.994) between PWF and TMP. As regards, NF is a pressure-driven procedure, transmembrane pressure (TMP) is a very crucial factor in the separation performance. Furthermore, as a constant-pressure procedure, TMP determines membrane permeability [30]. Accordingly the permeate flux is proportional to the TMP.

### Contact angle measurements

As the obtained results show, the highest water contact angle belongs to the unmodified PES membrane, which indicates the lowest hydrophilicity. A low contact angle with water indicates that the surface is hydrophilic. According the obtained result, the water contact angle on the PES membrane decreased from 74.7° to 28.3° once an optimal amount of Brij®58 surfactant was added to the casting solution. This may be attributed to the Brij®58 surfactant and morphology of the top and bottom surfaces of the membrane. As mentioned earlier, the PES membrane was modified by adding an optimal of Brij®58 to the casting solution. Therefore, changes in contact angle may relate to membrane modification. According to literature [31,32], the contact angle values depend on chemistry, roughness, and heterogeneity of surface as well as membrane parameters. This is consistence with the findings of the current study.

### Membrane surface charge

**Figure 5:**
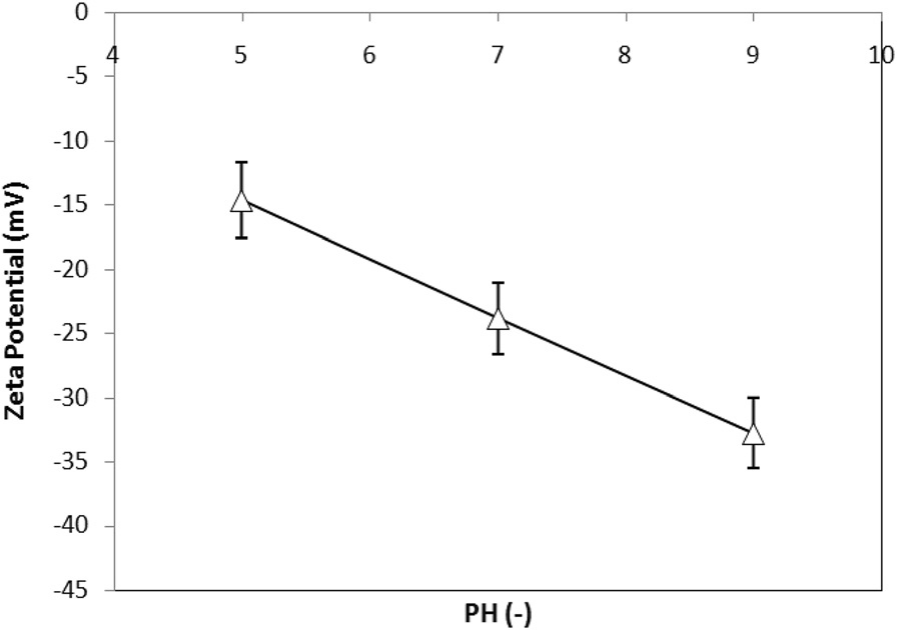
**Zeta potential of the self-made NF membrane as function of pH values.**

Zeta potential values presented in Figure 5 reveal negative charge of the NF membrane surface as well as a decrease in the absolute value of zeta potential at acidic pH. Zeta potential refers to surface charge that occurs in the presence of an aqueous solution when functional groups dissociate on surface or ions adsorb onto surface from the solution [33,34]. Since PES has no dissociated functional groups [35], specific ionic adsorption is the only possible process for the formation of surface charge.

### ATR-FTIR spectroscopy

**Figure 6:**
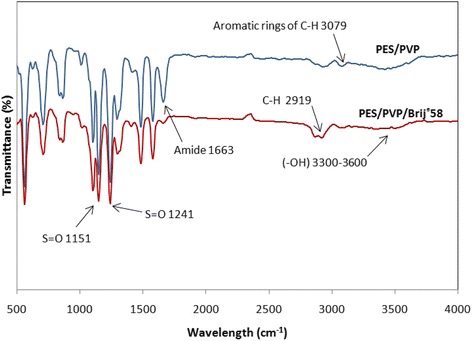
**ATR-FTIR spectra of the PES/PVP and PES/PVP/Brij®58 membranes.**

ATR-FTIR spectroscopy was used to ensure the presence of Brij®58 surfactant in the structure of the synthetic membrane during preparation process, especially in the coagulation stage. Figure 6 exhibits the surface ATR–FTIR spectra of the synthetic membrane. The functionalized self-made membrane illustrates four main peaks. The PES contains repeated ether and sulfone linkage alternating between aromatic rings. Accordingly, the bands at 1151 and 1241 cm^−1^ can be attributed to the stretching vibrations of S = O symmetric and S = O asymmetric, respectively. Besides, the bands at 1663, 3300–3600 and 2919 cm^−1^ depict the amide group of PVP, (−OH), and C–H groups of Brij®58 surfactant, respectively. However, the spectrum of PES/PVP/Brij®58 membrane shows that Brij®58 surfactant was retained in the membrane structure.

### Antibiotic rejection and permeation flux

**Figure 7:**
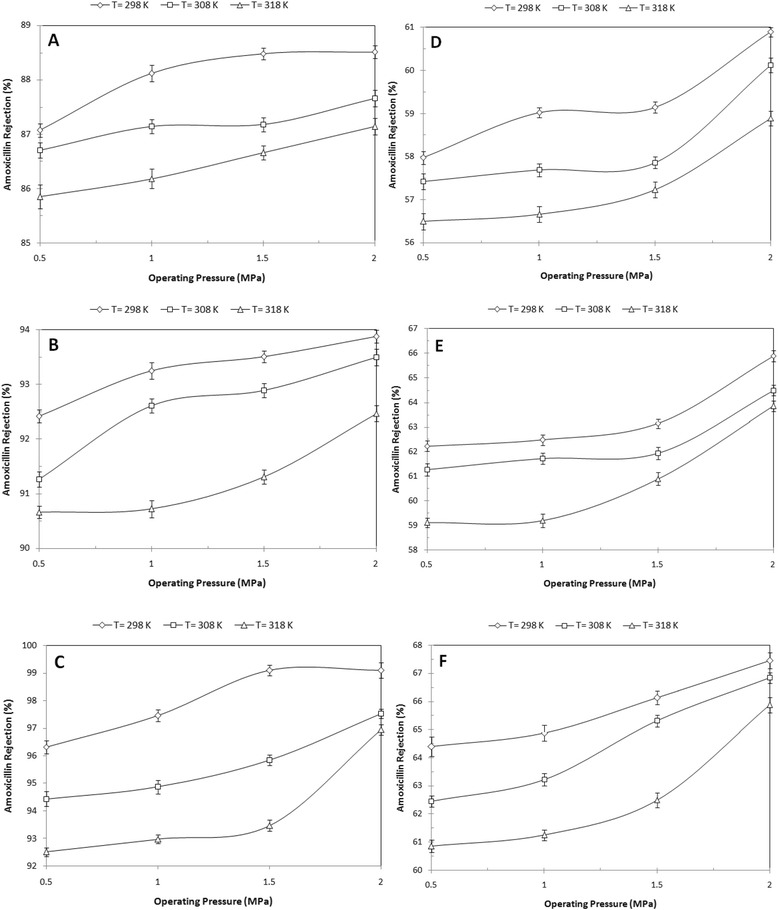
**Rejection of AMX vs. operating pressure at varying temperature and initial feed concentration. A)** 20 ppm/pH = 5.27, **B)** 20 ppm/pH = 7.0, **C)** 20 ppm/pH = 9.0, **D)** 400 ppm/pH = 5.01, E**)** 400 ppm/pH = 7.0, **F)** 400 ppm/pH = 9.0.

**Figure 8:**
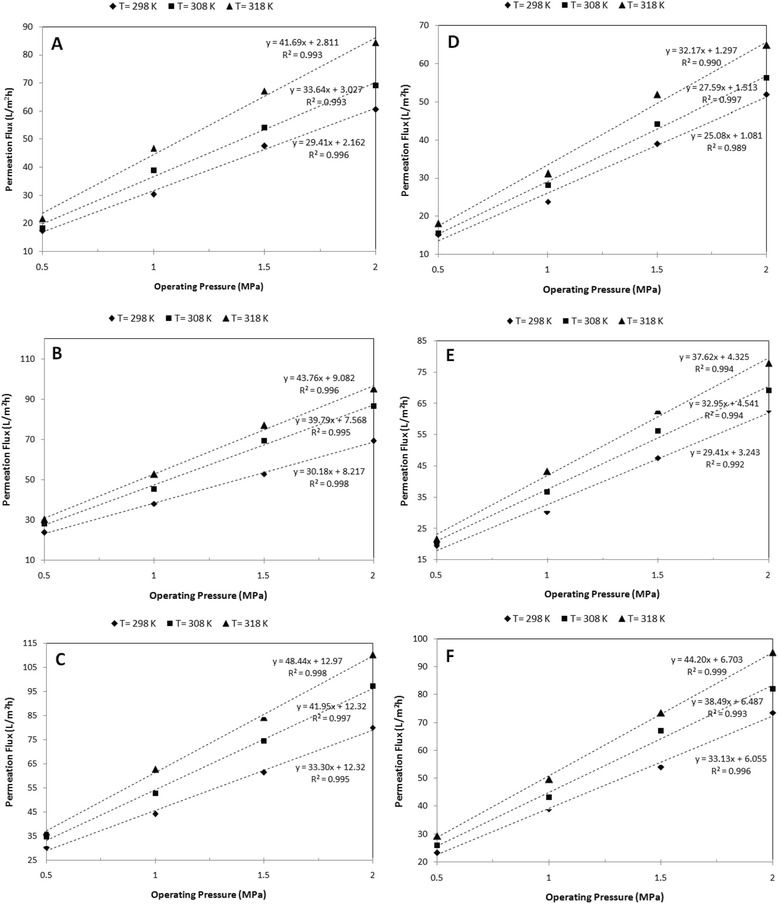
**Permeation Flux vs. operating pressure at varying temperature and initial feed concentration. A)** 20 ppm/pH = 5.27, **B)** 20 ppm/pH = 7.0, **C)** 20 ppm/pH = 9.0, **D)** 400 ppm/pH = 5.01, **E)** 400 ppm/pH = 7.0, **F)** 400 ppm/pH = 9.0.

The performance of the self-made membrane for the removal of AMX from wastewater is depicted in Figures 7 and 8. As the figures show, rejection and permeation flux by the self-made membrane varied from 56.49% to 99.09% and from15.14 L/m^2^h to 110.29 L/m^2^h, respectively. As shown by Figure 7, the effect of initial feed concentration on AMX rejection was much more than that of pH. Furthermore, the effect of operating pressure and temperature on the AMX rejection was noticeable.

According to Figure 8, the operating pressure, pH, and temperature have remarkable effects on the permeation flux.

The Pearson Correlation test results on AMX rejection and permeation flux are shown in Table 1. According to the results, the feed concentration has a significant effect on AMX rejection (P < 0.001) while it has no significant effect on the permeation flux. As such, AMX rejection decreased at high initial feed concentration of 400 ppm (Figure 7). This may be due to increased corresponding ionic strength that tends to neutralize the negative charges of the membrane and consequently, decrease electrostatic repulsion. Finally, a large number of ions pass through the membrane pores that result in a reduction in AMX rejection.

**Table 1:** **Pearson Correlation test for AMX rejection and permeation flux**

**Correlations**							
		**pH**	**Concentration**	**Pressure**	**Temperature**	**Rejection**	**Permeation flux**
**pH**	Pearson Correlation	1					
	Sig. (2-tailed)						
	R Square						
	N	72					
**Concentration**	Pearson Correlation	.000	1				
	Sig. (2-tailed)	1.000					
	R Square						
	N	72	72				
**Pressure**	Pearson Correlation	.000	.000	1			
	Sig. (2-tailed)	1.000	1.000				
	R Square						
	N	72	72	72			
**Temperature**	Pearson Correlation	.000	.000	.000	1		
	Sig. (2-tailed)	1.000	1.000	1.000			
	R Square						
	N	72	72	72	72		
**Rejection**	Pearson Correlation	.192	-.975^**^	.067	-.067	1	
	Sig. (2-tailed)	.105	.000	.575	.576		
	R Square	.037	.950	.005	.004		
	N	72	72	72	72	72	
**Permeation flux**	Pearson Correlation	.318^**^	-.211	.868^**^	.261^*^	.308^**^	1
	Sig. (2-tailed)	.007	.076	.000	.027	.008	
	R Square	.101	.044	.754	.068		
	N	72	72	72	72	72	72

**Correlation is significant at the 0.01 level (2-tailed).

*Correlation is significant at the 0.05 level (2-tailed).

Although, based on Figure 7, rejection rate of the AMX changed by pH changes, however, the impact of these changes is not noticeable according to Table 1. The results showed that increasing the pH from 5 to 9.0 increased rejection efficiency of AMX by 7%. AMX is an amphoteric substance with pKa_1_ = 2.4, pKa_2_ = 7.4, and pka_3_ = 9.6 [36]. AMX is zwitterions at medium pHs, a cation at pH = 2, and an anion at pHs above 7.4. Therefore, at higher pHs, AMX converts anionic forms. Surface charge of the self-made membrane becomes more negative with increasing pH (Figure 5). At a lower pH (5.27), molecular sieve mechanism dominates and results in medium rejection while at higher pH (9.0), Donnan repulsion mechanism involving electrostatic charge interactions between solute and membrane surface occurs. This leads to electrostatic repulsion between the AMX and self-membrane and higher membrane permeability (P < 0.001).

Significant effect of pH on permeability and AMX rejection by NF membrane has been confirmed by many researchers worldwide. As such, Derakhsheshpoor *et al*. investigated the effect of pH on the AMX rejection by high permeability polysulfone NF membrane. They observed that increasing feed pH from 6.3 to 8.3, improved 30% of AMX recovery [36].

Since NF is a pressure-driven process, increased pressure leads to an increase in permeate flux. According to Figure 8, the permeation flux increases with increasing operating pressure. This is due to the solution-diffusion model. According to Table 1, operating pressure has a significant effect on permeation flux (P < 0.01).

As Figure 7 suggests, increasing operating pressure from 0.5 to 2 MPa leads to an increase of approximately 3% in the AMX rejection. In other words, at high pressure, water permeability increases rapidly compared to the AMX by which greater number of water molecules can pass through the membrane. Based on Pearson Correlation test results (Table 1), the effect of operating pressure on AMX rejection is negligible.

Increased temperature leads to decreased AMX rejection (Figure 7) and increased permeation flux (Figure 8). This is due to the fact that increased temperature expedites thermal motion of molecules within the membrane, which contributes to the increase in the diffusion coefficient. Thus, transport of components is mainly controlled by the diffusion process in the membrane. In addition, the average pore size of the active separation layer increases slightly when the operating temperature goes up. It is in favor of an increase in permeation flux (P < 0.05).

Considering to Table 1, temperature change has no significant effect on the diffusion coefficient and rejection of AMX because the huge molar volume of AMX blocks the movement of AMX in the membrane.

## Conclusion

In this study, the self-made membrane was modified by a non-ionic surfactant (Brij®58). The self-made membrane was characterized by measuring zeta potential and contact angle. Modification of the membrane by Brij®58 surfactant was detected through ATR-FTIR spectroscopy. Moreover, the effect of operating conditions such as pH, feed concentration, operating pressure, and temperature on performance of the self-made membrane for removal of AMX from synthetic wastewater.

The results showed that adding an optimal amount of Brij®58 into the casting solution leads to a decrease in water contact angle. In other words, low water contact angle indicates that the membrane is hydrophilic. As the obtained results revealed, a increase in pH from 5 to 9.0 increases the permeation flux of approximately 18 L/m^2^h on average. Besides, an increase in pressure and temperature leads to increased permeation flux of the self-made membrane. The analysis results of the Pearson Correlation (Table 1) confirm that pH (P < 0.01), operating pressure (P < 0.01), and temperature (P < 0.05) have a significant effect on permeation flux, while operating pressure is not an effective parameter to improve the rejection efficiency. According to which, an increase in the operating pressure from 0.5 to 2 MPa increased the AMX rejection by approximately 3%. Moreover, pH and temperature have no significant effect on AMX rejection by the self-made membrane, as well.

Increased concentration adversely affects the efficiency of AMX rejection. In overall, maximum AMX rejection of 99.09% was achieved at operating temperature of 298 K, operating pressure of 2 MPa, initial feed concentration of 20 ppm, and pH of 9.0.

However, retention of organic pollutants in membrane separation process depends on the operating condition and characteristics of both membrane and pollutants. Generally, feed concentration is the only parameter affects adversely the rejection of AMX by self-made membrane. Considering AMX rejection efficiency of 99.09% by the self-made membrane at the initial feed concentration of 20 ppm, it can be concluded that this modified membrane is well suited for the removal of AMX from aquatic matrices containing low concentrations of this kind of pollutant. Other operating parameters such as temperature and pressure as well as the qualitative parameters of wastewater such as pH have negligible effect on AMX rejection by the self-made membrane. As a result, the self-made membrane is highly recommended under the above mention conditions.
